# Natural Products as Potentiators of β-Lactam Antibiotics: A Review of Mechanisms, Advances, and Future Directions

**DOI:** 10.3390/antiox15020154

**Published:** 2026-01-23

**Authors:** Wenjie Yang, Shuocheng Fan, Jie Luo, Yichu Zhou, Xingyang Dai, Jinhu Huang, Liping Wang, Xiaoming Wang

**Affiliations:** 1 Sanya Institute of Nanjing Agricultural University, College of Veterinary Medicine, Nanjing Agricultural University, Nanjing 210095, China; 9201710317@stu.njau.edu.cn (W.Y.); shuochengfan3@stu.njau.edu.cn (S.F.); luojie-@stu.njau.edu.cn (J.L.); 2024807224@stu.njeu.edu.cn (Y.Z.); t2024002@njau.edu.cn (X.D.); jhuang@njau.edu.cn (J.H.); 2 Department of Comparative Biosciences, University of Illinois at Urbana-Champaign, Urbana, IL 61802, USA

**Keywords:** natural products, β-lactam antibiotics, antibiotic resistance, synergistic effects, potentiators

## Abstract

This review focuses on the research progress on natural products as β-lactam antibiotic adjuvants, aiming to address the escalating challenge of antibiotic resistance, particularly the inactivation of antibiotics caused by β-lactamases. The article provides an in-depth analysis of the mechanisms by which plant-derived (e.g., flavonoids, tannins, phenolics, terpenoids, and alkaloids) and microbial-derived (e.g., clavulanic acid, fungal metabolites, bacteriophages) natural products enhance antimicrobial efficacy. Key potentiation strategies discussed include efflux pump inhibition, membrane permeability alteration, biofilm disruption, PBP2a inhibition, and direct β-lactamase inhibition. Additionally, the review outlines in vitro methods (e.g., dilution and checkerboard assays) and in vivo models (e.g., mouse infection models) used to assess synergistic effects. It also addresses major challenges in identifying active compounds, elucidating mechanisms of action, and pharmacokinetic characterization. Looking forward, the article highlights the potential of multi-omics approaches, artificial intelligence, and nanotechnology to overcome existing bottlenecks, providing novel strategies for the development of effective and safe antibiotic adjuvants. These advances are expected to provide both theoretical insights and practical guidance for combating antibiotic-resistant bacterial infections.

## 1. Introduction

Antibiotic resistance constitutes a significant global public health threat, with bacteria developing multiple mechanisms to evade the effects of antimicrobial agents [1]. β-lactam antibiotics, including penicillins, cephalosporins, carbapenems, and monobactams, are among the most extensively utilized and clinically effective classes of antibiotics [2]. In 2023, the global market for β-lactam and β-lactamase inhibitors was estimated at approximately USD 29 billion, representing nearly 57% of the total antibiotic market value, which stood at USD 50.91 billion [3]. These antibiotics exert their bactericidal activity by inhibiting bacterial cell wall synthesis. Specifically, they bind to penicillin-binding proteins (PBPs), enzymes responsible for cross-linking peptidoglycan, a crucial structural component of the bacterial cell wall [4]. This inhibition compromises cell wall integrity, resulting in osmotic imbalance and ultimately leading to bacterial cell lysis, making β-lactams exhibit broad-spectrum efficacy against diverse pathogens [5].

The therapeutic potential of β-lactam antimicrobial agents is increasingly compromised due to the global expansion of drug-resistant bacterial strains. A principal mechanism underlying this resistance is the bacterial production of β-lactamases—enzymes that hydrolyze the β-lactam ring, a four-membered lactam structure that constitutes the pharmacologically active core of these agents. Enzymatic cleavage of this moiety renders the antibiotic inactive [2]. This mechanism is particularly prevalent in Gram-negative bacteria, where β-lactamases are frequently encoded on mobile genetic elements, promoting horizontal gene transfer and rapid dissemination [6]. In addition to β-lactamases, bacteria have developed alternative resistance mechanisms, including mutations in PBPs (e.g., PBP2a in methicillin-resistant *Staphylococcus aureus*), which reduce β-lactam binding affinity, and the upregulation of efflux pumps that expel antibiotics from the intracellular environment. The emergence and global spread of multidrug-resistant (MDR) strains, resistant to three or more antibiotic classes—has further intensified this crisis, severely constraining available therapeutic options and posing a formidable challenge to public health systems worldwide.

The term ‘natural products’ refers to chemical compounds produced by living organisms. In the context of drug discovery, this term specifically denotes ‘secondary metabolites’. Unlike primary metabolites (e.g., amino acids and sugars) essential for survival, secondary metabolites—such as flavonoids, alkaloids, and terpenoids—play crucial roles in an organism’s defense, competition, and signaling. It is due to their vast chemical diversity and potent biological activities that natural products have long played a key role in drug discovery, serving as a prolific source of bioactive compounds, including antibiotics [7]. In recent years, increasing attention has been directed toward the use of natural products as potential adjuvants to β-lactam antibiotics, with the goal of restoring their efficacy against resistant bacterial strains [8]. These compounds can enhance β-lactam activity through diverse mechanisms, such as inhibiting β-lactamases, increasing bacterial membrane permeability, suppressing biofilm formation, and interfering with other resistance pathways [9].

Antibiotic resistance typically arises through genetic mutations or horizontal gene transfer, enabling bacteria to evade drug action via mechanisms such as target modification, enzymatic antibiotic degradation, reduced membrane permeability, or active efflux. These resistance traits are often rapidly selected under the strong selective pressure imposed by single-target, bactericidal antibiotics [10]. Natural products offer distinct advantages in combating resistance. Their vast chemical diversity provides a broad range of novel structures, which may reduce the likelihood of pre-existing bacterial resistance. However, it must also be considered that bacteria have long been exposed to these compounds in the natural environment, which may have facilitated the co-evolution of resistance mechanisms against certain natural products [11]. Nevertheless, many natural products exhibit multi-target or pleiotropic activity, simultaneously affecting multiple bacterial pathways. Such multi-site interference increases the evolutionary barrier to resistance by requiring concurrent adaptations at several loci, thereby reducing the probability of stable resistance emergence [12]. Clinically, the β-lactamase inhibitor clavulanic acid exemplifies the utility of natural products, having been effectively combined with β-lactam antibiotics such as amoxicillin [13]. Moreover, many natural products, particularly flavonoids and polyphenols, possess potent antioxidant properties that complement their antimicrobial activities [14]. These compounds can scavenge reactive oxygen species (ROS) generated during bacterial infections, thereby mitigating oxidative stress and preserving host tissue integrity. For instance, flavonoids like quercetin and epigallocatechin-3-gallate (EGCG) not only inhibit resistance mechanisms but also alleviate ROS-mediated cellular damage, thereby contributing to improved therapeutic outcomes in combination regimens [15]. Similarly, the coumarin derivative osthole has been reported to ameliorate myonecrosis caused by *Clostridium perfringens* infection via the activation of the Nrf2/HO-1 antioxidant pathway, effectively mitigating oxidative stress and inflammatory responses in host tissues [16]. Furthermore, derived from renewable sources, natural products present a sustainable and potentially cost-effective strategy for drug development.

This review explores recent advances in natural products as potentiators of β-lactam antibiotics, with a focus on their mechanisms of action, representative findings, and prospective research directions. A comprehensive literature survey was conducted using the PubMed database and the Web of Science citation index, covering studies published between 2015 and 2025. Search terms included “β-lactamase”, “synergy”, “natural products”, “synergistic effect”, “potentiators”, and “antibiotic synergism”.

## 2. Mechanisms of Natural Product-Mediated Potentiation of β-Lactam Antibiotics

**Figure 1:**
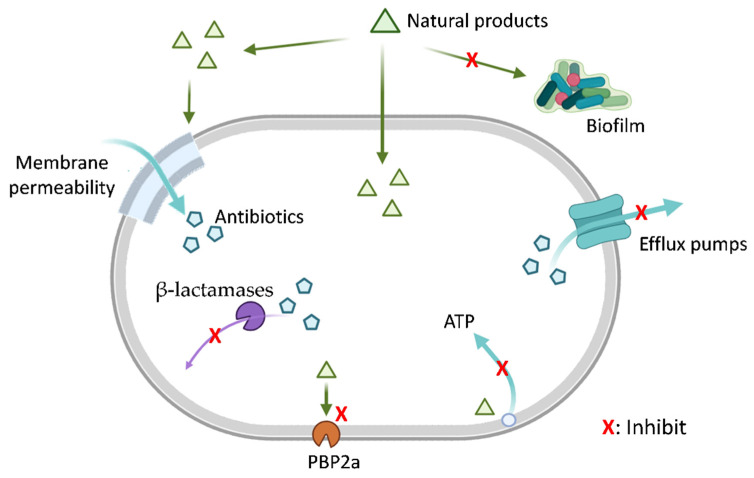
Natural Products Mitigate Bacterial Resistance Mechanisms. This schematic diagram illustrates the multifaceted strategies by which natural products (green triangles) may potentiate antibiotic activity by targeting bacterial resistance pathways. The diagram depicts mechanisms including inhibition of biofilm formation, alteration of bacterial membrane permeability, efflux pump inhibition, suppression of β-lactamase activity, inhibition of penicillin-binding protein 2a (PBP2a), and disruption of bacterial ATP generation (energy metabolism). Each red ‘X’ indicates a potential point of interference by natural products. Mechanisms are presented for conceptual completeness. Among them, β-lactamase inhibition and PBP2a inhibition are supported by substantial experimental evidence and, in some cases, clinical validation. In contrast, mechanisms such as modulation of ATP metabolism, membrane permeability, efflux activity, and biofilm-related processes are primarily supported by indirect or preclinical in vitro studies, and their roles in β-lactam potentiation remain less well established. Created in BioRender. Li, S. (2026) https://BioRender.com/c22k30u (accessed on 23 December 2025).

Natural products can enhance the efficacy of β-lactam antibiotics through various mechanisms, such as inhibiting drug efflux pumps, modulating cell membrane and wall permeability, disrupting biofilms, inhibiting PBP2a, suppressing β-lactamase activity, interfering with ATP synthesis, and other pathways (Figure 1).

### 2.1. Efflux Pumps Inhibition

Efflux pumps are transmembrane protein complexes that actively export a wide range of antibiotics out of the bacterial cell, thereby reducing intracellular drug concentrations and contributing to antimicrobial resistance [17]. This resistance mechanism is widely observed in both Gram-positive and Gram-negative bacteria, such as *Staphylococcus aureus*, *Escherichia coli*, and *Pseudomonas aeruginosa* [18].

Bacterial efflux pumps are primarily classified into several major superfamilies based on their structure and energy source [19]. Five of these are particularly prominent: the RND (Resistance Nodulation Division), MFS (Major Facilitator Superfamily), SMR (Small Multidrug Resistance), MATE (Multidrug and Toxic Compound Extrusion), and ABC (ATP-Binding Cassette) families [19]. ABC transporters utilize the energy from ATP hydrolysis, while the other four families are secondary transporters that rely on the proton motive force [20].

In Gram-negative bacteria, the RND family of efflux pumps is of paramount clinical importance due to its unique structure and high efficiency [19]. These pumps typically assemble into a tripartite complex that spans the inner membrane, the periplasm, and the outer membrane [20]. The AcrAB-TolC system in *E. coli* is a classic example, consisting of an inner membrane RND transporter (AcrB), a periplasmic adaptor protein (AcrA), and an outer membrane channel (TolC) [19]. This architecture allows the pump to expel captured drugs from the periplasm or inner membrane directly into the external medium, bypassing the periplasmic space and working synergistically with the low-permeability outer membrane barrier. In Gram-negative bacteria, this mechanism effectively protects targets on the inner membrane, such as PBPs, from antibiotics like many β-lactams that must first cross the outer membrane [18].

Natural products can act as Efflux Pump Inhibitors (EPIs) to subvert this defense system through several distinct mechanisms [17]. One primary mode of action is competitive inhibition, where the inhibitor acts as a substrate for the pump and competes with the antibiotic for the same binding site [17]. A more effective mechanism is often non-competitive or allosteric inhibition, where the inhibitor binds tightly to a distinct hydrophobic trap or “inhibitor-binding pit” within the pump protein [19]. This binding induces a conformational change that locks the pump in an inactive state, thereby blocking its functional rotation and transport cycle. Additionally, some compounds can indirectly inhibit proton-motive-force-dependent pumps by disrupting the proton gradient across the membrane, though this approach often lacks specificity and can be toxic to the cell [17].

Research has demonstrated that certain natural products are effective EPIs. For instance, EGCG enhances the susceptibility of *P. aeruginosa* to antibiotics like chloramphenicol and tetracyclines, likely by inhibiting the RND-family pump MexAB-OprM [15]. Similarly, berberine, an alkaloid found in traditional herbal medicines like *Corydalis* Tuber, has been identified as an efflux pump inhibitor in Gram-positive bacteria. Studies on *Staphylococcus aureus* demonstrated that berberine inhibits efflux activity, and molecular docking simulations suggest it binds to multiple efflux pump proteins, including MepA, NorA, NorB, and SdrM. This mechanism was shown to restore the efficacy of antibiotics such as ciprofloxacin and tobramycin against methicillin-resistant *S. aureus* (MRSA) [21]. These examples highlight the diverse strategies and potent potential of natural products to overcome efflux-mediated resistance, offering promising avenues for the development of combination therapies to re-sensitize resistant pathogens [17].

### 2.2. Alteration of Membrane and Cell Wall Permeability

The bacterial cell envelope, particularly the lipopolysaccharide (LPS)-rich outer membrane of Gram-negative bacteria, serves as a formidable permeability barrier that restricts the entry of many antibiotics [22]. Consequently, disrupting this barrier has emerged as a key therapeutic strategy to enhance antibiotic uptake and reverse resistance [22]. Natural products can function as permeability enhancers by directly interacting with membrane components, leading to destabilization, depolarization, and the formation of pores, which in turn facilitates the diffusion of antibiotics like β-lactams into the periplasm [13].

This principle is classically exemplified by polymyxins, such as colistin, which are natural peptides that specifically target the LPS of the Gram-negative outer membrane [23]. By electrostatically interacting with the anionic phosphate groups of lipid A, polymyxins competitively displace the divalent cations (Mg^2+^ and Ca^2+^) that stabilize the LPS layer [23]. This disruption damages the integrity of the outer membrane, making it more permeable and creating a synergistic effect that allows other antibiotics, such as β-lactams, to reach their periplasmic targets [24].

Many plant-derived compounds operate on similar principles. For instance, cinnamaldehyde enhances the susceptibility of multidrug-resistant *Salmonella* to ceftriaxone by causing cell membrane depolarization and observable physical damage, which is evidenced by increased LPS release [25]. Similarly, compounds like gallic acid (GA) and methyl gallate (MG) have been shown to induce significant morphological changes in the cell membrane of MRSA, including surface wrinkles and the leakage of intracellular contents [14]. Although the PBP targets in Gram-positive bacteria are located outside the cell membrane, this NP-induced membrane damage compromises the cell’s osmotic integrity, creating a synergistic lethal effect when combined with β-lactams that inhibit cell wall synthesis. While promising, membrane-targeting strategies also face challenges, notably ensuring selectivity for bacterial membranes over host cells to minimize toxicity, and the potential for bacteria to develop resistance by modifying their membrane structures, such as altering the net charge of their LPS [23].

### 2.3. Suppression of Biofilm Formation

Biofilms are structured bacterial communities embedded within a self-produced extracellular polymeric substance (EPS matrix composed of polysaccharides, proteins, and DNA). This matrix confers increased resistance by blocking antibiotic access to targets like PBPs and by harboring dormant bacteria that are less susceptible to cell wall inhibition [26,27]. Natural products can disrupt biofilms by inhibiting quorum sensing (QS), degrading the EPS matrix, or interfering with bacterial metabolism, thereby enhancing β-lactam efficacy.

Quorum sensing is a cell-to-cell communication system, mediated by signaling molecules, that coordinates gene expression and is crucial for biofilm maturation [28]. Natural products acting as QS inhibitors (QSIs) can block these signals, preventing mature biofilm formation and reducing resistance gene expression (e.g., *mecA*), thus increasing bacterial vulnerability. For example, baicalin synergizes with oxacillin against MRSA, lowering its minimum inhibitory concentration (MIC) four-fold, with a fractional inhibitory concentration index (FICI) ≤ 0.5, by inhibiting QS pathways, enhancing β-lactam penetration and bactericidal activity [29,30].

The EPS matrix acts as a physical shield, protecting embedded bacteria from antibiotics and host immune responses [27]. Degrading this layer with natural products, such as enzymes from bacteriophages, exposes bacterial targets. For instance, the lytic enzymes of bacteriophage F1Pa disrupt the EPS matrix of *Pseudomonas aeruginosa* biofilms, thereby facilitating piperacillin access to PBPs and resulting in a reduction in bacterial viability by over 2 log_10_ CFU/mL [31,32,33].

Bacteria within mature biofilms often enter a slow-growing or dormant metabolic state, rendering them tolerant to β-lactams that target actively dividing cells [26]. Natural products can disrupt this dormancy by interfering with essential metabolic processes and compromising membrane integrity. For instance, *Origanum vulgare* essential oil, rich in carvacrol, has been shown to damage the bacterial cell membrane, increase membrane permeability, and induce cytoplasmic leakage, as evidenced by potassium efflux, release of 260-nm–absorbing materials, and ultrastructural membrane alterations [34]. Such membrane disruption is widely associated with downstream energetic stress in bacteria, including impairment of cellular energy homeostasis and depletion of intracellular ATP, as reported for carvacrol and related essential oil components in independent metabolic studies [35]. This metabolic disruption reactivates dormant bacteria, enhancing their susceptibility to β-lactams. When combined with oxacillin, it reduces *S. aureus* biofilm biomass by approximately 50–60% (1–1.3 log_10_ CFU/mL) within 24 h, as shown by decreased viability [36]. Additionally, the oil induces reactive oxygen species (ROS) accumulation and membrane hyperpolarization, which may exacerbate metabolic stress, though the primary enhancement of β-lactam efficacy stems from ATP depletion and PBP targeting [35]. This synergistic action overcomes biofilm-mediated resistance, restoring bactericidal activity.

### 2.4. PBP2a Inhibition

Resistance to nearly all β-lactam antibiotics in MRSA is primarily mediated by the expression of Penicillin-Binding Protein 2a (PBP2a), an alternative transpeptidase encoded by the *mecA* gene [37]. Unlike the native PBPs of methicillin-susceptible *S. aureus* (MSSA), which are readily inactivated by β-lactams, PBP2a possesses a distorted active site that confers a low binding affinity for these antibiotics [38]. This structural distinction allows PBP2a to continue catalyzing the essential peptidoglycan cross-linking reactions required for cell wall biosynthesis, even when all other native PBPs are saturated by high concentrations of antibiotics, thereby enabling bacterial survival [37]. Therefore, inhibiting the function of PBP2a represents a critical strategy to overcome this resistance mechanism and restore the efficacy of β-lactam antibiotics against MRSA [39].

Natural products offer a diverse chemical arsenal to counteract PBP2a-mediated resistance through several distinct mechanisms, including the downregulation of gene expression, inhibition of protein translation, and direct binding to the enzyme [38]. For instance, the pomegranate-derived polyphenol punicalagin has been shown to suppress the transcription of the entire *mec* operon (*mecA*, *mecI*, and *mecR1*), leading to a significant reduction in PBP2a protein levels and restoring oxacillin susceptibility [40]. Similarly, glycyrrhizic acid nanoparticles (GA-NPs), formulated from the natural triterpenoid glycyrrhizic acid isolated from licorice, have been reported to potentiate the action of beta-lactams against MRSA by markedly suppressing PBP2a expression [41]. In this case, nanoparticle formulation primarily serves to improve the delivery and efficacy of the parent natural product, rather than representing a distinct class of antimicrobial agents.

Beyond transcriptional control, other natural compounds interfere at different stages of PBP2a production and function. Demethoxycurcumin (DMC), a natural curcuminoid from turmeric, hinders the translation of the PBP2a protein while also downregulating the transcription of its associated genes [37]. Furthermore, certain compounds exhibit multi-modal action; the non-membrane active antimicrobial peptide GN1, for example, resensitizes MRSA to β-lactams by both downregulating *mecA* gene expression and directly binding to an allosteric site on the PBP2a enzyme to inhibit its function [38]. In addition, a bioactive fraction from *Acalypha wilkesiana* has been shown to attenuate the production of PBP2a within the MRSA biofilm matrix [39].

Computational approaches have further expanded the list of potential PBP2a inhibitors. In silico molecular docking studies have predicted that flavonoids such as luteolin and kaempferol, as well as the microbial metabolite anisomycin, exhibit favorable binding affinity for PBP2a. Specifically, these compounds demonstrated calculated binding free energies of −8.47, −7.51, and −7.28 kcal/mol, respectively, which are comparable to or more negative than those of the reference antibiotic vancomycin (−6.95 kcal/mol) within the same docking framework [42]. These energetic profiles identify them as promising candidates for further experimental validation. Collectively, these findings demonstrate that targeting PBP2a with natural products—whether by suppressing gene expression, inhibiting protein synthesis, or through direct enzymatic binding—represents a highly promising and multifaceted strategy to overcome methicillin resistance and restore the clinical utility of β-lactam antibiotics against MRSA. It is essential to recognize, however, that most natural product–based strategies targeting the transcriptional or translational regulation of specific resistance genes, such as *mecA*, are currently restricted to the experimental or preclinical stages. To date, the clinical pharmacopoeia lacks approved beta-lactam adjuvants that primarily function through the selective modulation of resistance gene expression, emphasizing the forward-looking nature of these natural product investigations.

### 2.5. β-Lactamase Inhibition

The production of β-lactamase enzymes is the most significant mechanism of resistance to β-lactam antibiotics, particularly in Gram-negative bacteria [43,44]. These enzymes confer resistance by hydrolyzing the amide bond in the four-membered β-lactam ring, a core structural feature of all penicillins, cephalosporins, and carbapenems, thereby rendering the antibiotics pharmacologically inactive [6]. Based on amino acid sequence homology, β-lactamases are categorized into four molecular Ambler classes: A, B, C, and D [45]. This structural classification reflects fundamental mechanistic differences, as enzymes in classes A, C, and D utilize a serine residue in their active site for hydrolysis (serine β-lactamases), while class B enzymes are metallo-β-lactamases (MBLs) that require one or two zinc ions for their catalytic activity [44,45].

These enzymes present a formidable clinical challenge, especially those with broad hydrolytic spectrums that are encoded on mobile genetic elements, facilitating their rapid global dissemination [43]. Prominent examples include *Klebsiella pneumoniae* carbapenemase-2 (KPC-2), a class A serine carbapenemase, and New Delhi metallo-β-lactamase 1 (NDM-1), a class B metalloenzyme [43]. The widespread emergence of such enzymes, which can inactivate even last-resort carbapenem antibiotics, severely limits therapeutic options.

A clinically validated strategy to overcome this resistance is the co-administration of a β-lactamase inhibitor with a β-lactam antibiotic. Natural products serve as a rich source for such inhibitors, which can act by competitively or irreversibly binding to the enzyme’s active site. The archetypal example is clavulanic acid, a natural product derived from *Streptomyces clavuligerus*. As a potent inhibitor of many class A enzymes, clavulanic acid has been successfully developed for clinical use and is routinely co-administered with β-lactam antibiotics, such as in amoxicillin-clavulanate formulations [46]. Furthermore, crude soy saponins have been found to exhibit an inhibitory effect against various β-lactamases, including the challenging New Delhi metallo-β-lactamase 1 (NDM-1), with soyasaponin V identified as having the highest inhibitory activity against NDM-1. The synergistic effect on the antimicrobial activity of β-lactam antibiotics by crude soy saponins is thought to result from the inhibition of β-lactamase activity [47]. This approach of targeting β-lactamases with natural compounds offers a promising avenue to restore the effectiveness of existing β-lactam antibiotics and combat the growing threat of antibiotic resistance.

## 3. Advances in Natural Products as β-Lactam Antibiotic Potentiators

### 3.1. Advances in Research on Plant-Derived Natural Products

**Figure 2:**
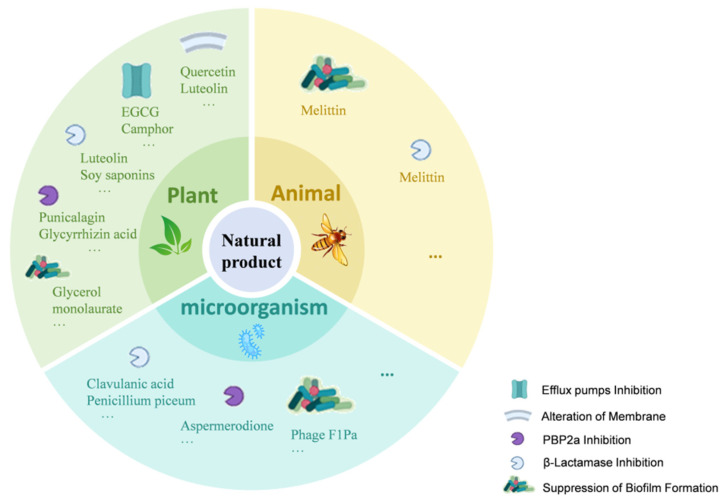
Mechanisms of Natural Products Enhancing β-Lactam Antibiotic Activity. This figure illustrates how natural products from plants, microorganisms, and animals enhance β-lactam antibiotic activity through various mechanisms. These include efflux pump inhibition (e.g., Quercetin, EGCG, Camphor); alteration of membrane permeability (e.g., Luteolin, Gallic acid, Eugenol); PBP2a inhibition (e.g., Punicalagin, Glycyrrhizin acid nanoparticles, Aspermerodione); β-lactamase inhibition (e.g., Soy saponins, Clavulanic acid); and suppression of biofilm formation (e.g., Glycerol monolaurate, Phage F1Pa, Melittin). The icons within the figure indicate the targets of these natural products, highlighting their multi-targeted strategies against bacterial resistance.

Plant-derived natural products possess rich chemical diversity, providing a broad candidate pool for the study of antibiotic potentiators [48]. These natural products enhance the antibacterial activity of β-lactam antibiotics through various mechanisms, including inhibiting efflux pumps, altering cell membrane and cell wall permeability, suppressing biofilm formation, inhibiting PBP2a, and suppressing β-lactamase activity (Figure 2). Key plant-derived natural products discussed in this section, their sources, representative structures, and primary mechanisms of potentiation are summarized in Table 1 (Plant-Derived section).

It should be noted that not all beneficial effects observed in combination studies constitute true pharmacological synergy or direct reversal of bacterial resistance. In particular, antioxidant and anti-inflammatory activities may improve therapeutic outcomes by mitigating host tissue damage or infection-associated stress but do not necessarily reflect direct modulation of bacterial resistance mechanisms.

#### 3.1.1. Flavonoids

Flavonoids, widely distributed in plant families such as Fabaceae, Lamiaceae, and Asteraceae, have attracted significant attention due to their antioxidant, anti-inflammatory, and antimicrobial properties. For instance, flavonoids such as quercetin and epigallocatechin gallate significantly enhance the antibacterial efficacy of β-lactam antibiotics against MRSA when administered in combination. The co-administration of quercetin (MIC > 512 μg/mL alone) and EGCG demonstrates potent synergistic antibacterial activity (FICI: 0.06–0.31), producing a bactericidal effect with up to a 4–5 log_10_ CFU/mL reduction in bacterial counts within 24 h [49], potentially by inhibiting resistance-related enzymes or enhancing membrane permeability. Quercetin exhibits antimicrobial activity and antioxidant properties that may help mitigate infection-associated oxidative stress, typically achieving a 4- to 32-fold reduction in the MICs of clinical antibiotics, thereby supporting improved therapeutic outcomes when used as an adjunct to β-lactam antibiotics [77]. EGCG, a principal polyphenolic constituent of green tea, exhibits robust antibacterial and antioxidant activities and enhances the efficacy of β-lactam antibiotics against carbapenem-resistant *Acinetobacter baumannii* by reducing MIC values (e.g., meropenem) from 64 μg/mL to 8–4 μg/mL (8- to 16-fold reduction; FICI ≤ 0.5), specifically by inhibiting multidrug efflux pumps and increasing intracellular antibiotic accumulation [78]. Its potent antioxidant activity neutralizes ROS, mitigating infection-induced oxidative damage and contributing to enhanced antibacterial efficacy in combination with β-lactam antibiotics. Similarly, theaflavin-3,3′-digallate (TF3), a polyphenol from black tea, significantly enhances the antibacterial activity of carbapenems against NDM-1-producing Enterobacteriaceae by decreasing the MIC of meropenem from 64 μg/mL to 4 μg/mL (16-fold reduction; FICI ≤ 0.25), through directly inhibiting metallo-beta-lactamase activity. This compound demonstrates robust synergistic effects both in vitro and in a murine pneumonia model, effectively reducing bacterial load and improving survival rates [50]. Luteolin has also exhibited synergistic activity with ampicillin and oxacillin against MRSA, potentially by enhancing membrane permeability or downregulating the expression of resistance genes [51]. Other flavonoids, including galangin [52], baicalin [29], and liquiritigenin [53], have also been shown to potentiate β-lactam antibiotics through diverse mechanisms, providing a broad spectrum of plant-derived candidates for combating resistant bacteria.

#### 3.1.2. Tannins

Tannins, a class of polyphenolic compounds widely distributed in plants, possess notable astringent and antibacterial properties and can effectively counteract bacterial resistance. Tannic acid, primarily derived from leguminous plants, has been reported to sensitize MRSA to β-lactam antibiotics, effectively reducing the MICs of agents such as oxacillin and ampicillin by 2- to 16-fold (with synergistic FICI values 0.174–0.477) when used at sub-inhibitory concentrations, likely through interference with cell wall synthesis or resistance gene expression [54]. Additionally, its antioxidant properties reduce ROS-mediated oxidative stress, contributing to improving therapeutic outcomes when combined with β-lactam antibiotics. Punicalagin, a key tannin compound found in pomegranates, reverses MRSA resistance to oxacillin by significantly decreasing the MIC from 256 μg/mL to 16 μg/mL (16-fold reduction; FICI = 0.31) through the inhibition of mecA and PBP2a expression [40]. It has also been demonstrated to potentiate the activity of β-lactams against MDR *Enterobacteriaceae*, where its combination with amoxicillin/clavulanic acid resulted in an increase of up to 54% in the growth inhibition area against *Salmonella typhi* isolates [55]. These findings highlight the potential of tannin-based compounds as adjunctive therapies to enhance β-lactam efficacy and provide novel strategies for overcoming bacterial resistance.

#### 3.1.3. Phenolic Compounds

Phenolic compounds, recognized for their antibacterial and antioxidant properties, enhance the efficacy of β-lactam antibiotics by disrupting bacterial membranes or inhibiting resistance-associated enzyme activity. Cinnamic acid and its derivatives (such as ferulic acid and p-coumaric acid), in combination with ampicillin and cefazolin, exhibit synergistic effects against *Staphylococcus epidermidis* (FICI ≤ 0.5), possibly by compromising membrane integrity or increasing antibiotic permeability [56]. Gallic acid and its methyl ester enhance the antibacterial activity of ampicillin and penicillin G against MRSA, with in vitro studies and molecular docking analyses suggesting β-lactamase inhibition as a potential mechanism [14]. Additionally, its antioxidant effects diminish ROS accumulation during bacterial infections, augmenting the antibacterial efficacy of β-lactam antibiotics. Eugenol, a phenolic compound derived from cloves, has demonstrated synergistic bactericidal activity with cefotaxime against *S. aureus*, reducing the antibiotic’s MICs by at least 4-fold (FICI = 0.375), likely by increasing membrane permeability and facilitating antibiotic uptake [57]. Cinnamaldehyde enhances ceftriaxone activity against MDR *Salmonella* by significantly decreasing the MIC from 128 μg/mL to 8 μg/mL (16-fold reduction; FICI = 0.375), primarily through downregulating extended-spectrum β-lactamase (ESBL) expression and disrupting bacterial membranes [25]. Moreover, glycerol monolaurate, when combined with ampicillin, exerts synergistic effects against MRSA with a 4- to 16-fold reduction in MIC values (FICI: 0.15–0.5), highlighting the broad applicability of phenolic compounds as β-lactam adjuvants [58].

#### 3.1.4. Terpenoids

Terpenoids, primarily derived from Lamiaceae and Zingiberaceae plants, possess antibacterial and anti-inflammatory activities. These compounds have been shown to act synergistically with β-lactam antibiotics by increasing membrane permeability or inhibiting resistance mechanisms. Camphor, a monoterpenoid present in *Mentha* and *Thymus* species, reduces the MIC of carbapenem-resistant *Acinetobacter* spp. when combined with imipenem, achieving synergistic effects with FICI values as low as 0.37 and reducing the antibiotic’s MIC by up to 4-fold, potentially by enhancing membrane permeability or inhibiting efflux pumps [59,62]. 1,8-Cineole, in combination with amoxicillin, significantly enhances efficacy against ESBL-producing *E. coli* and *K. pneumoniae*, reducing the antibiotic’s MIC from 512 μg/mL to as low as 64 μg/mL (8-fold reduction; FICI ≤ 0.5), primarily by inhibiting enzymatic resistance mechanisms [60]. Carvacrol, another monoterpenoid, exhibits synergistic antibacterial activity against *E. coli* when combined with cefixim, demonstrating a synergistic FICI of 0.5, likely by disrupting the bacterial membrane and increasing antibiotic penetration [61]. Additionally, the clerodane diterpenoid 12(S), 16S-dihydroxycleroda-3,13-diene-15,16-olide, isolated from *Justicia* species, enhances the susceptibility of MRSA to β-lactam antibiotics, reducing the MIC of oxacillin from 16 μg/mL to 0.5 μg/mL (32-fold reduction; FICI = 0.125), demonstrating a remarkable synergistic effect [62].

#### 3.1.5. Alkaloids

Alkaloids have demonstrated potentiation of beta-lactam antibiotics against MDR pathogens. Berberine, a protoberberine alkaloid, has surfaced as a promising antibiotic adjuvant. Experimental evidence indicates that berberine hydrochloride significantly restores the susceptibility of MDR *Acinetobacter baumannii* to carbapenems, reducing the MIC of meropenem from 64 μg/mL to 8 μg/mL (8-fold reduction; FICI = 0.375) [79]. It has also been reported to synergize with other beta-lactams against MRSA by inhibiting efflux pump systems and disrupting bacterial metabolic processes [63].

#### 3.1.6. Other Classes of Phytochemicals

Beyond the major classes of natural products, a variety of plant-derived natural products, including saponins, lignans, and polyphenols, synergize with β-lactam antibiotics through various mechanisms, offering diverse options for antibacterial therapy. Saponin compounds, such as ginsenoside Rg3 and soybean saponins derived from soybeans, have demonstrated synergistic antibacterial activity by enhancing the sensitivity of MRSA to oxacillin and cefazolin, and by inhibiting β-lactamases such as NDM-1, respectively [47,64]. Glycyrrhizin nanoparticles reduce MRSA activity by inhibiting PBP2a [41]. Lignan compounds, including honokiol, exhibit synergistic antibacterial effects with β-lactam antibiotics against MRSA and vancomycin-resistant enterococci (VRE), potentially by disrupting cell division through interference with the FtsZ protein [65]. Polyphenol compounds, such as cranberry proanthocyanidins (cPAC), restore the activity of oxacillin against MRSA by inhibiting ESBLs and MBLs, thus overcoming PBP2a-mediated resistance [66]. Lignin, a primary component of plant cell walls, also restores MRSA susceptibility to β-lactam antibiotics when used in combination with oxacillin, by disrupting cell wall structure and resulting in ultrastructural disorganization [67]. Other phytochemicals, such as fucoxanthin and hypericin [68], exert their effects by enhancing ceftriaxone activity or inhibiting the expression of resistance genes, respectively. The structural and functional diversity of these compounds expands the repertoire of synergistic adjuvants and provides valuable leads for the development of novel antibacterial interventions.

### 3.2. Advances in Research on Microbial-Derived Natural Products

Microbial sources, including bacteria and filamentous fungi, have long been an essential font of antibiotics and their adjuvants. These sources produce small-molecule natural products that exhibit significant potential in combating resistant bacterial strains through mechanisms such as β-lactamase inhibition, enhanced targeting of cell wall synthesis, and inhibition of biofilm formation (Figure 2). In addition, other biological entities, such as bacteriophages, have emerged as powerful synergistic agents that operate through distinct mechanisms. Prominent examples are compiled in Table 1 (Microbial-Derived section).

#### 3.2.1. Small-Molecule Potentiators from Bacteria and Fungi

This section focuses on small-molecule compounds produced by bacteria and filamentous fungi (e.g., *Penicillium*, *Aspergillus*), which act as sources of antibacterial agents, distinguishing them from pathogenic yeasts such as *Candida albicans*.

Actinomycetes are a crucial source of β-lactamase inhibitors. Clavulanic acid, produced by *Streptomyces clavuligerus*, remains the archetypal example [80]. It is a broad-spectrum, mechanism-based inhibitor that irreversibly inactivates many class A serine β-lactamases. Clinically, its combination with amoxicillin (e.g., Augmentin) is a cornerstone therapy for infections caused by β-lactamase-producing pathogens, and recent advancements in genetic and metabolic engineering continue to improve its biosynthetic yield [81].

Beyond clavulanic acid, other historically significant inhibitors have been discovered. The olivanic acids, a family of natural carbapenems also from *Streptomyces* species, demonstrated potent, broad-spectrum inhibition of both class A and C enzymes [82]. A mechanistically distinct molecule, lactivicin, isolated from *Lysobacter* and *Empedobacter* species, represented a paradigm shift; though it is not a β-lactam, its novel bicyclic structure mimics the transition state of β-lactam hydrolysis, enabling it to acylate and inhibit both PBPs and certain serine β-lactamases [83]. The discovery of these compounds broadened the chemical scaffolds known to inhibit β-lactamases and provided key insights for the development of modern synthetic inhibitors [84].

The fungal kingdom, particularly the genera *Penicillium* and *Aspergillus*, has also demonstrated strong potential. While the *Penicillium* genus is famed for producing penicillin, its potential as a source of β-lactamase inhibitors remains underexplored, though some metabolites from *Penicillium piceum* show inhibitory activity [69]. The *Aspergillus* genus, however, has yielded notable potentiators targeting different resistance pathways. Aspergillomarasmine A (AMA), a natural product from *Aspergillus versicolor*, acts as a potent MBL inhibitor. Recent efficacy studies demonstrate that the addition of AMA (8–16 μg/mL) effectively rescues the activity of meropenem against NDM-1-producing *Klebsiella pneumoniae*, restoring bacterial susceptibility to clinical breakpoints [70]. In contrast, aspermerodione, a metabolite from an endophytic *Aspergillus* sp., targets MRSA by binding to an allosteric site on PBP2a; it has been shown to reduce the MIC of oxacillin against MRSA ATCC 43300 from 128 μg/mL to 2–4 μg/mL(up to a 64-fold reduction) [71]. Fungi also produce antimicrobial peptides (AMPs), such as plectasin, a defensin that exhibits synergistic bactericidal activity against MRSA when combined with penicillins [73].

Other microbial ecosystems and compounds also serve as rich sources of adjuvants. Humimycin 17S, a lipopeptide from the human microbiome, shows potent synergy with ampicillin against MRSA and VRE [74]. Metabolites from probiotics like *Enterococcus faecium* can act as quorum-sensing inhibitors, reducing MRSA resistance to cefoxitin and attenuating its virulence [75]. Lastly, tunicamycin (TUN) and its less cytotoxic synthetic derivatives, such as TunR1, enhance beta-lactam activity by inhibiting phosphoglycosyl transferases. In models using *Bacillus subtilis*, TunR1 was found to potentiate the activity of various beta-lactams, including oxacillin and cephalosporins, by factors of up to 256-fold [72].

#### 3.2.2. Bacteriophages as Synergistic Agents

Distinct from small-molecule natural products, bacteriophages—viruses that specifically infect bacteria—represent a powerful biological strategy to enhance antibiotic efficacy. Their synergy often stems from their ability to physically dismantle bacterial defenses, most notably biofilms. For instance, phage F1Pa exhibits synergistic activity with β-lactam antibiotics against multidrug-resistant *P. aeruginosa* by disrupting the biofilm matrix, which allows the antibiotic to reach its target cells [33]. This approach physically removes the protective barrier, restoring antibiotic susceptibility. Phage therapy combined with ceftaroline has also shown synergistic bactericidal activity against daptomycin-resistant VISA and MRSA strains while inhibiting biofilm formation [85]. Other studies have confirmed that bacteriophages and their lytic proteins enhance the activity of cephalosporins and piperacillin against pathogens like *Shigella dysenteriae* and *P. aeruginosa* PAO1 [86,87]. While the high specificity of phages offers a favorable safety profile by avoiding off-target effects, it also presents a challenge for broad-spectrum applications, and the potential for phage resistance remains an area of active investigation.

This growing body of research highlights the immense potential of products from diverse microbial sources as β-lactam antibiotic potentiators. Further exploration of these compounds and biological entities could provide novel strategies for overcoming antibiotic resistance.

### 3.3. Research Status and Limitations of Animal-Derived Natural Products

In contrast to plant- and microbial-derived natural products, research on animal-derived natural products remains relatively scarce. Current studies mainly focus on AMPs, such as melittin from bee venom, which have demonstrated certain antibacterial activities. However, their potential as β-lactam antibiotic adjuvants remains underexplored, and the available data are insufficient to support their widespread use. Moreover, animal-derived natural products face challenges such as difficulty in extraction, low yield, and complex composition. Mechanistic understanding and comprehensive safety evaluations are also lacking, further hindering their development in this field. Future research should focus on screening, isolating, and elucidating the mechanisms of animal-derived natural products to explore their potential as antimicrobial adjuvants.

AMPs are important components of animal-derived natural products and typically exert their effects by disrupting bacterial membranes, inhibiting bacterial metabolism, or interfering with biofilm formation. Among them, melittin—a cationic AMP derived from bee venom—has demonstrated synergistic antibacterial activity when combined with β-lactam antibiotics such as penicillin and oxacillin against MRSA and VRSA. In vitro experiments indicated that melittin can disrupt bacterial biofilms, thereby enhancing the bactericidal efficacy of β-lactam antibiotics [76].

In addition to AMPs, certain structural proteins and their derivatives from animals have also been found to possess antibacterial properties, though research on their role as β-lactamase inhibitors or adjuvants remains in its infancy. The antimicrobial protein Seroin, found in silkworm silk, has shown antibacterial activity, particularly against Gram-positive bacteria. Current research mainly focuses on its antimicrobial mechanisms and structural modifications, but no direct evidence has yet confirmed its role as a β-lactam antibiotic adjuvant [88].

It is important to acknowledge that many of the membrane-disrupting or membrane-active natural products discussed in this section possess the potential to enhance the activity of various antibiotic classes, rather than acting strictly as beta-lactam-specific adjuvants. Nevertheless, considering the paramount clinical significance of beta-lactams and the pivotal role that membrane permeability plays in their resistance mechanisms, this review maintains a dedicated focus on their combinational efficacy with this class of antibiotics.

## 4. Research on the Combined Application of Natural Products and β-Lactam Antibiotics

### 4.1. Screening Methods for Natural Products as Antibiotic Potentiators or Inhibitors

The escalating challenge of antibiotic resistance necessitates the discovery of natural products that can act as potentiators to enhance the efficacy of β-lactam antibiotics against MDR bacteria [89]. High-throughput screening (HTS) and virtual screening (VS) represent two complementary discovery strategies widely employed to identify candidate β-lactam potentiators. At this early stage, the primary objective is to efficiently prioritize active candidates from large chemical or natural product libraries for downstream mechanism and pharmacological evaluation.

**Figure 3:**
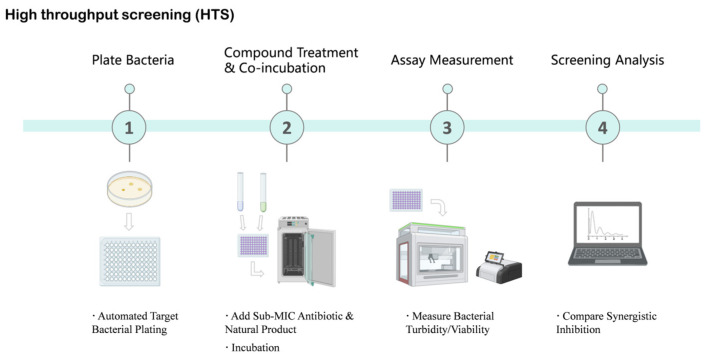
High-Throughput Screening (HTS) Workflow for Identifying Natural Product Antibiotic Potentiators. This schematic diagram illustrates the key steps involved in high-throughput screening to identify natural products that enhance antibiotic efficacy. The process begins with automated target bacterial plating, followed by the addition of sub-minimum inhibitory concentration (MIC) antibiotics and natural products for incubation. Subsequently, bacterial turbidity or viability is measured using automated readers. Finally, screening analysis is performed to compare synergistic inhibition effects, aiming to identify promising natural product hits. Created in BioRender. Li, S. (2026) https://BioRender.com/bpw3b6c (accessed on 17 January 2026).

HTS-based discovery of β-lactam potentiators typically relies on synergy-oriented phenotypic assays or direct enzyme inhibition formats, enabling the rapid experimental evaluation of thousands to hundreds of thousands of compounds [90,91]. For example, a fluorescence-quenching assay based on the hydrolysis of the chromogenic substrate nitrocefin was used to screen nearly 190,000 compounds against the OXA-48 β-lactamase, leading to the identification of novel inhibitor scaffolds such as the pyrazolo[3,4-b]pyridine core [91]. Similarly, a near-infrared fluorescent probe (LXMB) was developed to selectively detect β-lactamase (Blac) activity from *Mycobacterium tuberculosis* and applied to the screening of herbal medicines, resulting in the identification of tannic acid as a Blac inhibitor that enhanced the activity of multiple β-lactam antibiotics against *M. tuberculosis* H37Ra (Figure 3) [92].

When applied to natural products (NPs), however, HTS campaigns face distinct physicochemical challenges that differ from those encountered with conventional synthetic libraries. Many NPs, including polyphenols, terpenoids, and fatty acid derivatives, exhibit limited aqueous solubility and therefore require the use of organic solvents such as DMSO or surfactants (e.g., Tween 80) for screening. Importantly, accumulating evidence indicates that solvent composition itself may influence bacterial growth, membrane integrity, or biofilm formation, underscoring the necessity of rigorous vehicle controls to avoid false-positive potentiation effects during synergy screening [93,94]. These considerations are particularly relevant during primary HTS, where subtle solvent-dependent artifacts may be amplified across large datasets.

**Figure 4:**
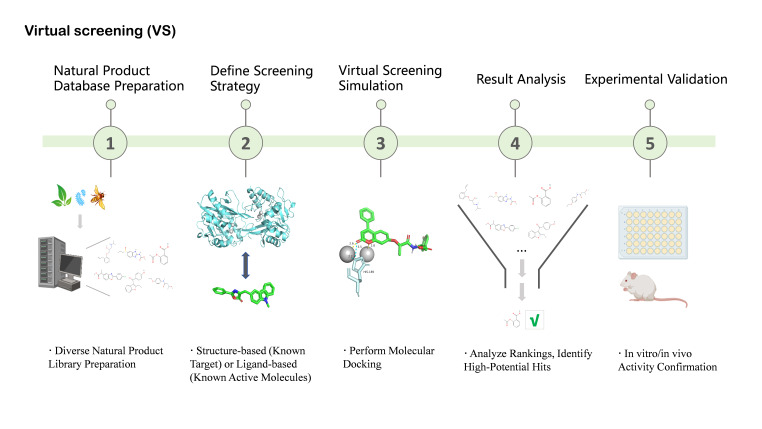
Virtual Screening (VS) Workflow for Natural Product Discovery. This schematic diagram outlines the key steps involved in leveraging virtual screening to identify potential natural product hits. The process commences with diverse natural product library preparation, followed by defining the screening strategy (either structure-based using a known target or ligand-based using known active molecules). Subsequently, molecular docking is performed to predict binding or activity. This leads to result analysis and ranking to identify high-potential “hit” molecules. Finally, these selected candidates undergo in vitro/in vivo activity confirmation. Created in BioRender. Li, S. (2026) https://BioRender.com/xlvn6h2 (accessed on 17 January 2026).

VS serves as a complementary in silico strategy that is especially valuable for prioritizing NP-derived candidates prior to experimental validation. Structure-based VS has been successfully applied to clinically relevant β-lactam resistance targets, including serine- and metallo-β-lactamases as well as altered penicillin-binding proteins (PBPs) [90,95]. For instance, large-scale VS campaigns have identified inhibitors of carbapenemases such as GES-5 by screening extensive libraries of drug-like compounds [90]. Parallel VS against multiple resistance enzymes, including KPC-2 and the metallo-β-lactamases NDM-1 and VIM-2, has further enabled the identification of broad-spectrum inhibitor scaffolds [89]. Mutated PBPs, most notably PBP2a in MRSA, have also emerged as important VS targets for rational discovery efforts [96] (Figure 4). Nevertheless, the application of VS to natural products presents additional challenges arising from their structural complexity, conformational flexibility, and frequent deviation from conventional “drug-like” chemical space. These features can complicate docking accuracy and scoring reliability, necessitating careful target selection, appropriate constraint strategies, and experimental validation to minimize false positives. Accordingly, the integration of HTS and VS—coupled with NP-specific controls and prioritization criteria—provides a pragmatic and efficient framework for the early-stage discovery of natural product–derived β-lactam potentiators.

### 4.2. Methodological Adaptations for In Vitro Evaluation of Natural Product–Based Potentiators

Following the identification of candidate β-lactam potentiators, rigorous in vitro evaluation is essential to accurately assess antibacterial activity, quantify synergistic interactions, and assign bioactivity to specific compounds. Standard in vitro assays—such as MIC determination, checkerboard assays, and time-kill studies—remain foundational tools for evaluating antibiotic potentiation [97]. However, when applied to natural products, these methods require careful methodological adaptation to account for compound-specific properties that may confound assay readouts and data interpretation.

One major challenge in the in vitro evaluation of NP-based potentiators is interference with conventional optical or colorimetric readouts. Many plant-derived NPs, including polyphenols, flavonoids, and tannins, are intrinsically pigmented or redox-active and can artificially influence optical density (OD)–based growth measurements in microdilution assays. To mitigate such interference, modified protocols incorporating metabolic indicators such as resazurin have been widely adopted, enabling indirect assessment of bacterial viability through redox-dependent fluorescence or color change rather than turbidity alone [98,99]. These approaches have proven particularly valuable for evaluating crude extracts, essential oils, and antimicrobial peptides, although confirmatory CFU-based assays are often required to validate apparent synergistic effects.

Volatility represents an additional NP-specific consideration that is not adequately captured by standard liquid-phase in vitro assays. Volatile compounds, such as essential oils and low-molecular-weight terpenoids, may partially evaporate during incubation, leading to underestimation of antibacterial or potentiating activity in broth-based MIC or checkerboard formats. In such cases, vapor-phase or modified diffusion-based assays—such as inverted Petri dish or sealed-chamber systems—provide a more appropriate framework for evaluating antimicrobial efficacy and synergy under conditions that better reflect their physicochemical behavior [100,101]. These approaches are particularly relevant for assessing NP–β-lactam combinations intended for respiratory or topical applications.

A further methodological challenge arises from the chemical complexity of many NP preparations. Unlike single-molecule synthetic compounds, NPs are frequently encountered as partially characterized extracts or heterogeneous mixtures containing dozens to hundreds of constituents. In this context, activity-guided fractionation approaches, including thin-layer chromatography (TLC)–bioautography, offer an effective strategy for localizing antibacterial or potentiating activity directly on chromatographic media [102,103]. By coupling chemical separation with biological detection, TLC-bioautography facilitates the rapid identification of active fractions prior to full structural elucidation, thereby reducing the risk of misattributing activity to inactive or minor components.

Collectively, these methodological adaptations highlight the importance of tailoring in vitro evaluation strategies to the unique properties of natural products. Integrating standard synergy assays with NP-specific controls, alternative readouts, and activity-localization techniques enhances the reliability and interpretability of in vitro data, providing a more robust foundation for downstream mechanistic studies and in vivo validation of β-lactam potentiators.

### 4.3. Models for In Vivo Validation of Natural Product–Based β-Lactam Potentiators

Following promising in vitro results, in vivo validation represents a critical step in assessing the translational potential of natural product–based β-lactam potentiators. Animal infection models provide an integrated physiological context in which antibacterial efficacy, pharmacokinetics, toxicity, and host–pathogen interactions can be evaluated simultaneously. Commonly employed models include murine skin, pulmonary, and systemic infection models, which remain essential for establishing in vivo synergy and therapeutic relevance [104]. Additionally, the Caenorhabditis elegans infection model provides a high-throughput and ethically advantageous bridge between in vitro assays and mammalian studies for early-stage efficacy and β-lactam potentiation screening [105]; however, its limited pharmacokinetic resolution necessitates subsequent validation in higher vertebrate models to confirm translational relevance.

When applied to NPs, however, vivo evaluation presents additional challenges that extend beyond those encountered with conventional small-molecule antibiotics. A major limitation arises from unfavorable pharmacokinetic (PK) properties, including poor solubility, low oral bioavailability, rapid metabolism, and limited tissue penetration. These factors can lead to discrepancies between in vitro potency and in vivo efficacy, complicating the interpretation of synergy observed in animal models. Consequently, careful PK characterization and dose optimization are essential to distinguish true pharmacological potentiation from exposure-limited efficacy [104].

Route of administration constitutes another NP-specific consideration in in vivo validation. While many β-lactam antibiotics are administered systemically, certain classes of NPs—such as essential oils or highly lipophilic compounds—may be more effectively delivered via alternative routes, including topical, inhalational, or localized administration. For example, inhalation-based infection models or aerosolized delivery systems may be more appropriate for evaluating volatile or respiratory-targeted NP–β-lactam combinations, whereas topical wound infection models may better capture the therapeutic potential of membrane-active or poorly absorbed compounds [100].

In addition, host-mediated effects must be carefully considered when interpreting in vivo outcomes for NP-based potentiators. Many natural products possess anti-inflammatory or antioxidant activities that can modulate disease severity, tissue damage, or immune responses independently of direct antibacterial action. While such effects may contribute to improved therapeutic outcomes, they do not necessarily reflect direct reversal of bacterial resistance mechanisms. Therefore, in vivo studies should integrate bacterial burden measurements (e.g., CFU reduction) with host-response markers to clearly delineate antibacterial potentiation from host-directed benefits [106].

Overall, in vivo validation of natural product–derived β-lactam potentiators requires tailored experimental design that accounts for NP-specific pharmacokinetic constraints, delivery strategies, and host effects. Integrating appropriate animal models with PK-informed dosing and mechanistic endpoints enhances the translational relevance of preclinical findings and supports more informed progression toward clinical development.

## 5. Challenges and Prospects in Natural Product Research

### 5.1. Challenges

The chemical complexity of natural products complicates the identification of active components that enhance antibacterial effects. Plant extracts contain numerous compounds with intricate interactions, requiring the integration of multiple chromatographic techniques (such as silica gel column and reversed-phase C18 column chromatography), high-performance liquid chromatography (HPLC), gas chromatography (GC), mass spectrometry (MS), and nuclear magnetic resonance (NMR) spectroscopy for repeated separation and purification. This process is time-consuming, yields low quantities of target compounds, and carries a high risk of component inactivation. Additionally, microbial metabolites are influenced by strain variability and culture conditions, leading to unstable production and structural diversity. The lag in new metabolite identification technologies further restricts the discovery and development of antibacterial potentiating compounds, consequently impeding progress in novel antimicrobial drug research [107].

The mechanisms of natural products involve multiple targets and pathways, rendering their elucidation particularly complex. Although certain studies have made progress, most remain confined to the cellular level, lacking detailed molecular-level characterization. Key knowledge gaps include the dynamic interactions between natural compounds and bacterial targets, the complexity of regulatory signaling networks, and an insufficient understanding of the deep molecular mechanisms underlying bacterial resistance reversal. Despite the generation of massive datasets through multi-omics approaches, the integration and interpretation of this information remain major obstacles, substantially limiting the comprehension of antibacterial potentiation mechanisms and impeding drug design, development, and innovation [108].

Pharmacokinetic studies of natural products face multiple difficulties. Their absorption is constrained by chemical structure, physicochemical properties, and gastrointestinal physiological conditions. For example, flavonoids and similar compounds possess high molecular weights and strong polarity, often undergoing glycosylation modifications that hinder absorption and reduce bioavailability. Studies on tissue distribution lack highly sensitive detection methods and reliable in vivo models. The metabolic pathways of natural products are highly complex, involving diverse enzymatic reactions, which complicates the identification of metabolite activity and potential toxicity. Additionally, research on pharmacokinetic–pharmacodynamic (PK-PD) correlations of natural products remains insufficient. Excretion profiling is further complicated by the structural complexity of natural products, while the lack of sensitive detection technologies constrains dosage form development and clinical application, ultimately hindering their clinical translation and broader therapeutic use [109,110].

The chemical complexity, mechanistic diversity, and pharmacokinetic challenges of natural products vary significantly across their structural and functional categories. Structurally, natural products can be classified into flavonoids (e.g., quercetin, EGCG), phenolics (e.g., gallic acid, cinnamaldehyde), terpenoids (e.g., carvacrol), alkaloids (e.g., berberine), microbial metabolites (e.g., clavulanic acid), and AMPs (e.g., melittin). Flavonoids, due to their high polarity and glycosylation, require complex chromatographic techniques (e.g., HPLC, C18 columns), which are time-consuming and risk activity loss [9]. Microbial metabolites face production instability due to strain variability and culture conditions, necessitating optimized fermentation processes [81]. AMPs are limited by low yields and extraction difficulties [76]. Mechanistically, β-lactamase inhibitors (e.g., soy saponins) have well-defined targets, whereas efflux pump inhibitors (e.g., berberine) involve complex dynamic interactions requiring advanced molecular imaging [17]. Pharmacokinetically, flavonoids and alkaloids suffer from low bioavailability largely due to their high polarity and associated poor solubility/absorption profiles [111,112], while terpenoids’ high volatility and sensitivity to environmental factors (such as light, heat, and oxygen) compromise formulation stability and shelf-life [113]. These category-specific challenges underscore the need for tailored technological solutions.

### 5.2. Future Prospects

Based on the current body of evidence, different classes of natural products exhibit markedly distinct potentials for clinical translation as β-lactam antibiotic potentiators. Microbial-derived metabolites, particularly β-lactamase inhibitors and PBP-targeting compounds, currently represent the most clinically promising category, as exemplified by clavulanic acid and aspergillomarasmine A, which demonstrate well-defined mechanisms, robust in vivo efficacy [4,114], and, in some cases, established clinical use. These compounds typically act on specific and validated resistance targets, making their pharmacological optimization and regulatory development more feasible.

In contrast, many plant-derived natural products, including flavonoids, phenolics, and terpenoids, display broad-spectrum potentiating effects through mechanisms such as membrane disruption, efflux pump modulation, and biofilm suppression. While these compounds offer valuable chemical diversity and multifunctional biological activities, their clinical translation is often constrained by issues related to bioavailability, target specificity, and pharmacokinetic variability. Consequently, further structural optimization, formulation strategies, and in vivo validation are required before these agents can be advanced toward clinical application [115].

Collectively, these observations suggest that microbial-derived natural products with defined resistance targets are currently best positioned for near-term clinical development, whereas plant-derived compounds may serve as important long-term leads or adjunctive scaffolds pending further optimization.

Despite these differences in translational readiness, both microbial- and plant-derived natural products face common challenges, including incomplete mechanistic understanding, limited target validation, and insufficient pharmacokinetic characterization. Addressing these barriers requires systematic, mechanism-driven approaches that can more precisely link natural product activity to bacterial resistance pathways. To this end, the development of novel antibacterial potentiators can be facilitated by multi-omics technologies, including genomics, transcriptomics, proteomics, and metabolomics, to construct comprehensive molecular interaction networks of natural products within bacterial systems. This strategy enables the precise identification of critical targets and signaling pathways involved in antibacterial potentiation, thereby offering a clear framework for drug discovery and development [116]. Additionally, advances in artificial intelligence (AI) facilitate in-depth data mining and support virtual screening coupled with experimental validation. Such approaches significantly accelerate lead compound optimization, reduce development costs, and enhance research efficiency and success rates [117]. Furthermore, advancements in microfluidic chip technology have led to the development of miniaturized and integrated analytical platforms. These platforms enable rapid analysis of small samples, accurately simulate in vivo microenvironments, and allow for in-depth studies of natural product–bacteria interactions and pharmacokinetics, thereby introducing innovation into natural product research and advancing antimicrobial drug development [118]. Moreover, leveraging the antioxidant properties of natural products presents promising avenues for combating antibiotic resistance. By integrating multi-omics to identify the molecular targets of antioxidant compounds and applying nanotechnology to enhance their bioavailability, researchers can develop β-lactam potentiators that concurrently target bacterial infections and oxidative stress, providing novel therapeutic strategies for clinical applications.

Natural products exhibit substantial potential as β-lactam antibiotic potentiators in clinical settings. For instance, the combination of clavulanic acid and amoxicillin (Augmentin) has been clinically effective against infections caused by β-lactamase-producing bacteria, illustrating the practical value of this strategy. Other natural compounds, such as malleamycin A [70], have demonstrated promising preclinical efficacy by restoring bacterial susceptibility to β-lactam antibiotics. However, the clinical translation of these compounds faces several obstacles, including toxicity, side effects, dosage safety, and unfavorable pharmacokinetic properties. Many natural compounds exhibit low bioavailability, making it difficult to reach effective concentrations in vivo. Moreover, the absence of large-scale clinical trial data significantly restricts their widespread adoption. Future research should focus on conducting more preclinical and clinical studies, particularly human trials, to confirm the safety and therapeutic efficacy of these natural compounds.

Nanotechnology offers a promising strategy to overcome the pharmacokinetic limitations of natural compounds. The application of nanocarriers, such as nanoparticles and liposomes, enhances drug stability, facilitates targeted delivery, enables controlled release, and improves antibacterial potentiation while minimizing adverse effects. These advancements contribute to improved patient adherence and overall treatment outcomes [119,120].

In addition, the establishment of a clinical monitoring system for antibacterial potentiators derived from natural products is crucial. A comprehensive assessment of their efficacy, safety, and resistance characteristics, combined with real-time treatment adjustments informed by monitoring data, will support rational clinical application. Such a strategy will facilitate the efficient translation of natural product research from bench to bedside, thereby contributing to the fight against antibiotic resistance and the protection of global public health.

As discussed above, natural products exhibit substantial structural and functional diversity, which underlies their varied mechanisms of β-lactam potentiation. This diversity is reflected in the markedly different translational trajectories observed across major natural product classes. Microbial metabolites, such as clavulanic acid and aspergillomarasmine A, exhibit the highest potential due to their specific β-lactamase inhibition and proven clinical success (e.g., Augmentin), with multi-omics and genetic engineering enhancing yield and target identification [46,70]. Phenolics (e.g., cinnamaldehyde) and terpenoids (e.g., carvacrol), with simple structures and broad-spectrum activity, are ideal for rapid development as topical or local adjuvants, leveraging nanotechnology to improve stability [25,61]. Flavonoids (e.g., EGCG) provide notable anti-inflammatory benefits that may support improved therapeutic outcomes when used alongside β-lactam antibiotics, but their clinical translation is often limited by low bioavailability, necessitating AI-driven structural optimization [78]. Alkaloids (e.g., berberine) target complex resistance but face toxicity challenges [63]. AMPs, despite novel mechanisms, are limited by extraction costs [76]. Prioritizing microbial metabolites and accelerating phenolics and terpenoids via emerging technologies will drive innovative β-lactam potentiator development.

## 6. Conclusions

Significant progress has been made in the investigation of natural products as β-lactam antibiotic potentiators, offering a promising strategy to combat antibiotic resistance. Natural products derived from plants and microbes enhance the antibacterial activity of β-lactam antibiotics through diverse mechanisms, including efflux pump inhibition, increased membrane permeability, and direct β-lactamase inhibition. Representative compounds, such as clavulanic acid and malleamycin A, have demonstrated substantial value in both clinical applications and research. Both in vitro and in vivo experimental models provide reliable means for evaluating their synergistic antibacterial effects.

Nonetheless, several challenges persist, including difficulties in identifying active components, incomplete elucidation of mechanisms of action, and insufficient pharmacokinetic studies. While an increasing repertoire of phytochemicals has been identified as β-lactam potentiators, the field is still constrained by a paucity of translational studies bridging molecular discovery and therapeutic application. Bridging this gap will require a multidisciplinary approach integrating pharmacokinetic optimization, target-based screening, and structure-guided drug design, ultimately enabling the development of next-generation β-lactam adjuvants derived from natural scaffolds.

In summary, current evidence indicates that distinct classes of natural products differ substantially in their translational potential as β-lactam antibiotic potentiators. Microbial-derived metabolites, particularly β-lactamase inhibitors and penicillin-binding protein (PBP)-targeting compounds, emerge as the most compelling candidates for near-term clinical translation, supported by well-defined mechanisms of action, robust in vivo validation, and, in some cases, established clinical use. By contrast, many plant-derived natural products are better positioned as long-term leads, as further optimization is often required to overcome limitations related to bioavailability, pharmacokinetics, and target specificity.

Building on these translational priorities, future research integrating multi-omics approaches, artificial intelligence, and nanotechnology holds great promise for precisely identifying active compounds, elucidating their mechanisms of action, and optimizing their pharmacokinetic properties. These advancements are expected to accelerate the development of novel antibacterial potentiators and provide innovative strategies to combat resistant bacterial infections. Ultimately, these efforts will contribute to optimizing clinical antibacterial therapy, offering broad applications and substantial public health benefits.

An important but relatively underexplored question concerns the potential development and frequency of bacterial resistance to natural product–based antibiotic adjuvants. At present, systematic data on the emergence or prevalence of resistance to NP adjuvants remain limited, with most available evidence derived from short-term in vitro studies. Because many NP adjuvants act by targeting resistance mechanisms, stress response pathways, or membrane integrity rather than essential bacterial growth processes, they may impose different selective pressures compared with conventional antibiotics. Nevertheless, adaptive responses, compensatory mechanisms, or cross-resistance cannot be excluded, underscoring the need for long-term evolutionary and in vivo studies.

## Figures and Tables

**Table 1:** Classification of Natural Product-Derived beta-Lactam Potentiators by Biological Origin. Mechanisms marked with an asterisk (*) indicate those categorized as “Validated,” supported by direct biochemical evidence, genetic assays, and/or in vivo studies. Unmarked mechanisms are classified as “Postulated,” primarily derived from in vitro phenotypic observations (e.g., checkerboard assays) and require further mechanistic confirmation. To ensure clarity within the table, evidence levels are integrated via these symbols. Chemical structures are standard 2D representations sourced from authoritative databases (e.g., PubChem). For heterogeneous mixtures or broad compound classes (e.g., soy saponins, cranberry proanthocyanidins, or essential oils), characteristic scaffolds or representative bioactive monomers are depicted for illustrative purposes and do not represent a single defined molecular entity.

Natural Product	Source (Origin)	Structure	Mechanism	Reference
Plant-Derived				
Quercetin	Ubiquitous in plants	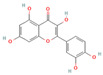	Enhance membrane permeability	[49]
Epigallocatechin-3-gallate (EGCG)	*Camellia sinensis* (green tea)	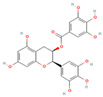	Inhibits/interferes with efflux pumps (e.g., MexAB-OprM) *	[15]
Theaflavin-3,3′-digallate	*Camellia sinensis* (black tea)	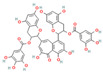	Binds PBP2a allosteric site; inhibits metallo-β-lactamases *	[50]
Luteolin	Ubiquitous in plants	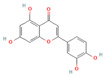	Synergy via membrane disruption or resistance gene suppression *	[51]
Galangin	*Alpinia officinarum* (Zingiberaceae)	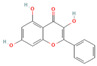	Enhances β-lactam activity	[52]
Baicalin	*Scutellaria baicalensis*	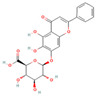	Disrupts bacterial membrane integrity (depolarization and leakage) and inhibits biofilm formation *	[29]
Liquiritigenin	*Glycyrrhiza glabra* (Fabaceae)	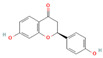	Reverses resistance by acting synergistically with beta-lactams *	[53]
Tannic acid	Leguminous plants	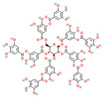	Sensitizes MRSA to β-lactams	[54]
Punicalagin	*Punica granatum* (Lythraceae)	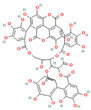	Sensitizes pathogens by inhibiting efflux pumps, and damaging cell membrane integrity *	[55]
Cinnamic acid derivatives	*Cinnamomum* spp. (Lauraceae)	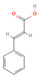	Acts synergistically to potentiate beta-lactam activity	[56]
Gallic acid	*Quercus* spp., *Camellia sinensis*	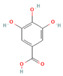	Modulates membrane permeability; putative β-lactamase inhibition	[14]
Eugenol	*Syzygium aromaticum* (Myrtaceae)	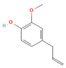	Increases membrane permeability	[57]
Cinnamaldehyde	*Cinnamomum* spp. (Lauraceae)	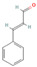	Disrupts membranes; Inhibits efflux pump; Disrupts energy metabolism *	[25]
Glycerol monolaurate	Coconut oil derivatives	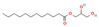	Enhances betalactam efficacy *	[58]
Camphor	*Cinnamomum camphora* (Lauraceae)	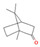	Reduces carbapenem MIC via efflux inhibition	[59]
1,8-Cineole	*Eucalyptus* spp. (Myrtaceae)	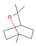	Potentiates beta-lactams by directly inhibiting ESBL enzymatic activity and increasing bacterial membrane permeability *	[60]
Carvacrol	Oregano, thyme (Lamiaceae)	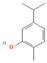	Membrane disruption; Enhances antibiotic penetration *	[61]
Clerodane diterpenoid	*Callicarpa americana* (Lamiaceae)	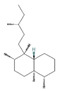	Restores β-lactam susceptibility in MRSA *	[62]
Berberine	*Berberis* spp. (Berberidaceae)	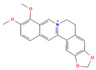	Inhibits efflux pumps; metabolic interference *	[63]
Ginsenoside Rg3	*Panax ginseng* (Araliaceae)	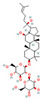	Membrane depolarization and increased permeability *	[64]
Soy saponins	*Glycine max* (Fabaceae)	-	Inhibits β-lactamases (including NDM-1) *	[47]
Glycyrrhizin acid	Chinese herb licorice	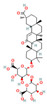	Suppress PBP2a *	[41]
Honokiol	*Magnolia officinalis* (Magnoliaceae)	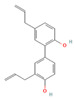	Inhibits bacterial cell division by directly binding to the FtsZ protei, suppressing its GTPase activity and polymerization, while also disrupting membrane integrity *	[65]
Cranberry proanthocyanidins	*Vaccinium macrocarpon* (Ericaceae)	-	Inhibits ESBL/MBL enzymes *	[66]
Lignin	Plant cell walls	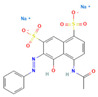	Disrupts cell envelope homeostasis by damaging the cell membrane *	[67]
Fucoxanthin	Brown algae (Phaeophyceae)	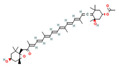	Enhances ceftriaxone efficacy	[68]
Hypericin	*Hypericum perforatum* (Hypericaceae)	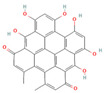	Suppresses sarA-mediated resistance regulation *	[68]
*Origanum vulgare* essential oil	Oregano plant	-	Disrupts cell membrane *	[34]
Microbial-Derived				
Clavulanic acid	*Streptomyces clavuligerus*	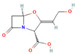	Irreversible inhibition of β-lactamases *	[46]
*Penicillium piceum*	Penicillium genus	-	Inhibits β-Lactamase *	[69]
Aspergillomarasmine A	*Aspergillus versicolor*	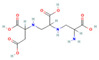	Potent metallo-β-lactamase (MBL) inhibitor *	[70]
Aspermerodione	*Aspergillus* sp. TJ23 (endophytic fungus)	-	Binds PBP2a allosteric site; inhibits MRSA resistance *	[71]
Tunicamycin (TUN) derivatives	*Streptomyces lysosuperificus*	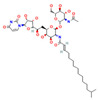	Acts as a non-toxic potentiator by inhibiting the bacterial wall teichoic acid (WTA) synthesis pathway *	[72]
Plectasin	Fungal defensin peptide (saprophytic fungi)	-	Enhances antibiotic activity by binding to the cell wall precursor Lipid II to inhibit peptidoglycan biosynthesis *	[73]
Humimycin 17S	Human microbiome-derived lipopeptide	-	Inhibits the Lipid II flippase MurJ, thereby disrupting the transport of cell wall precursors and compromising cell wall integrity *	[74]
Probiotic metabolites	*Enterococcus faecium*, *Lactococcus lactis*	-	Reduces MRSA resistance via quorum sensing inhibition *	[75]
Phage F1Pa	Bacteriophages	-	Disrupts biofilms; enhances antibiotic penetration *	[33]
Animal-Derived				
Melittin	*Apis mellifera* (honeybee venom)	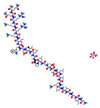	Disrupts the bacterial cell membrane (increasing permeability) and degrades the protective biofilm matrix *	[76]

## Data Availability

No new data were created or analyzed in this study. Data sharing is not applicable to this article.

## Abbreviations

The following abbreviations are used in this manuscript:

AMPs	Antimicrobial peptides
CFU	Colony-forming units
EGCG	Epigallocatechin-3-gallate
ESBLs	Extended-spectrum β-lactamases
FICI	Fractional inhibitory concentration index
MBLs	Metallo-β-lactamases
MDR	Multidrug resistance
MIC	Minimum inhibitory concentration
MRSA	Methicillin-resistant Staphylococcus aureus
PBPs	Penicillin-binding proteins
PBP2a	Penicillin-binding protein 2a
PK/PD	Pharmacokinetics/pharmacodynamics
QS	Quorum sensing
ROS	Reactive oxygen species
EPS	Extracellular polymeric substance
